# Assessment of iodine concentration in dietary salt at household level in Morocco

**DOI:** 10.1186/s12889-016-3108-8

**Published:** 2016-05-20

**Authors:** Ahmed Zahidi, Meriem Zahidi, Jamal Taoufik

**Affiliations:** Department of Drug Sciences, Laboratory of Medicinal Chemistry, Faculty of Medicine and Pharmacy, Mohammed V University in Rabat, P. O. Box 6203, Rabat_Institutes Rabat, Morocco

**Keywords:** Dietary sodium chloride, Iodized salt, Iodine, Potassium iodate, Households, Morocco

## Abstract

**Background:**

Following WHO recommendations, Morocco adopted in 1995 the universal salt iodization (USI) as a strategy to prevent and control iodine deficiency disorders. In 2009, the standard salt iodine concentration was adjusted to 15–40 mg/kg. The success of USI for the control of iodine deficiency disorders requires an evaluation of iodine concentration in salt prior to assessing the iodine nutritional status of a population.

**Methods:**

In our study we refer to the anterior studies that were made in Morocco in 1993 and 1998. 178 salt samples from households were tested for iodine using spot-testing kits. The iodometric titration method was used to analyze accurately the concentration of iodine in the 178 household salt samples. An empiric polling method was adopted, using a non-probability sampling method; across the different twelve regions in the country.

**Results:**

The median and interquartile range iodine concentration in salt was 2.9 mg/kg (IQR: 2.4-3.7). The results show that only 25 % of households use iodized salt. The recommended iodine concentration in salt of 15–40 mg/kg was met only in 4.5 % of salt samples. The bulk salt is used by 8 % of households. All samples of this bulk salt were found in rural areas. According to nonparametric appropriate tests used, there is no significant difference in iodine concentrations between regions, between urban and rural areas and between packaged and bulk salt.

**Conclusions:**

Two decades since introducing legislation on Universal Salt Iodization, our survey shows that generalization of iodized salt is far from being reached. In 2015, only a quarter of Moroccan households use the iodized salt and only 4.5 % of salt is in conformity with regulations. The use of bulk salt by households in rural areas constitutes a major obstacle to the success of USI. The National Iodine Deficiency Disorders Control Program can only be achieved if an internal follow-up and a control of external quality of program is put in place.

## Background

Iodine is a trace element present in human body at very low quantities (10-15 mg). The only known role of iodine in the human body is to constitute an essential element in the synthesis of the thyroid hormones: Thyroxin and Triiodothyronine (T3) [1].

In case of iodine deficiency, anomalies in thyroid function and goiter may occur; they vary according to the relevance of iodine deficiency and age of subjects. These sets of complications are known under the term of Iodine Deficiency Disorders (IDD) [2].

Iodine deficiency holds a notorious place among the micronutrient deficiencies which now pose a major worldwide public health problem as it is the leading cause of both thyroid and brain abnormalities in children [3]. It is especially the first cause of thyroid disorders and cerebral abnormalities in children. So, it leads to a slowing of all metabolic functions and the psychomotor alteration. It is the first cause of avoidable mental retardation in worldwide [3].

Iodine deficiency disorders stands as a major obstacle to economic development, due to the severity of irreversible neurological damage caused, it constitutes a factor of social and economic exclusion [4, 5]. It is estimated that in the general population, 2 billion people have insufficient iodine intake which 31.5 % of school-age children [6]. 38 million children born every year at risk of brain damage because of iodine deficiency [7]. For the elimination of iodine deficiency, WHO recommended Universal Salt Iodization [8], this includes how should be used by all members of the population after 1 year of age [9], which corresponds to an intake of < 5 g of salt per day according to WHO recommendations on sodium intake [10].

In India, the national average of self reported consumption is 76.1 %, but only 51.1 % of Indians consume adequately iodized salt 2005–2006 [11].

Morocco is considered as a moderate iodine deficiency area. A survey in 1993 found that 63 % of children aged 6 to 12 years had urinary iodine less than normal (<100 μg/L) and 22 % had a goiter [12]. Following WHO recommendations, Morocco has adopted the universal salt iodization strategy, in order to prevent and control iodine deficiency disorders. Thus mandatory iodization of table salt at 80 ± 10 mg of iodine/kg was introduced in 1995 and subsequently adjusted to a level of 15–40 mg of iodine/kg in 2009 [13]. Numerous studies have been conducted to evaluate the iodine status in many parts of Morocco [14–16].

To assess the success of Moroccan salt iodization program, it is critical to control iodine concentration and iodized salt coverage, which must be done before checking the iodine nutritional status of the population [17].

## Methods

### Sample design

The study is a cross-sectional study; it covered all of the 12 Moroccan regions according to the administrative division of February 2015. For sampling, we adopted the empirical method of non-probability sampling across the different regions. The convenience sampling was used taking into account results of two previous studies with similar objective where the rate of iodized salt was 9.8 % and 15 % respectively [16, 18].

The sample size is calculated using the non-probability sampling with 1.96 for a confidence level of 0.95 and absolute error risk of 5 %. According to data above the minimum sample size to be considered is 167.

### Data collection

To reach the different 12 regions of Morocco, we have chosen a number of pharmacy students, friends, acquaintances and family members to which we explained our study, and who were willing to bring us salt samples used in their households or households of their parents.

**Fig. 1:**
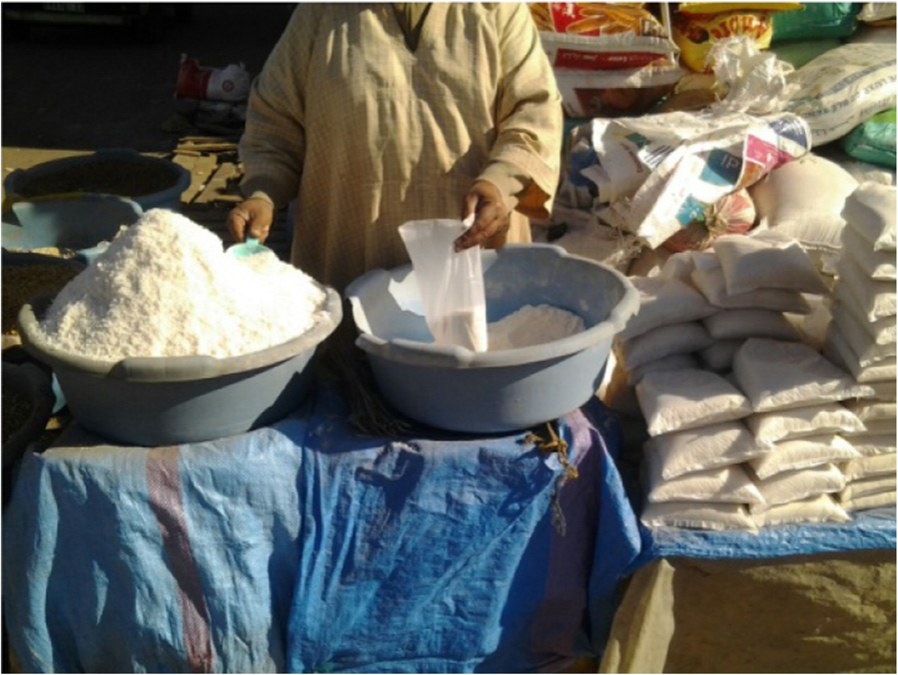
Bulk salt for retail sale at traditional market in Marrakech-Safi region

Samples were collected during the period October 13, 2014 to March 30, 2015. The samples were coded and stored at room temperature and protected from light and moisture until the time of analysis. Sampling sheet was filled in order to identify the address of the household, province, urban or rural areas, the type of salt (packaging salt or bulk salt sold in traditional markets) (Fig. 1) and the information mentioned on packets of salt (Labeling iodized salt; Logo representative of iodized salt; mentioning quantity of iodine added; mentioning having sanitary authorization; mentioning having ONSSA authorization; Date of manufacture/expiry; Address of the company).

All the samples were analyzed by the same person using the spot testing kits (MBI KITS International how is provided by the management of Epidemiology and the fight against non-transmissible diseases) [19] and iodometric titration procedure [8, 20] in the Laboratory of Medicinal Chemistry, Faculty of Medicine and Pharmacy of Rabat.

### Validation of spot-testing kits to determine iodine content in salt

The spot-testing kit used to determine the percentage of salt samples with iodine concentration above and below 15 ppm (mg/kg of salt) [19, 21]. It gives a qualitative estimation of the iodine content. Salt samples are classified as iodized or non-iodized. Depending on the intensity of the color obtained, the test gives an indication of the level of iodine in the salt. If the test showed “no iodine” on the first testing, the check solution was added again. This was required to neutralize the presence of alkali in salt and to acidify the salt sample. If the test showed no iodine when tested for a second time, this was taken as the negative test result [19].

### Measurement of iodine concentration in salt by iodometric titration procedure

Iodometric titration consists of a preparation of reagents followed by two analytical steps. In these two steps, the free iodine is released from salt and the free iodine is then titrated with thiosulfate using starch as an external indicator. The results are expressed as milligram of iodine per kilogram of salt (mg/kg) [20].

### Data analysis

Basic descriptive statistics were calculated, including frequencies, proportions, means, and medians. For Categorical data, the percentages and number of samples in each category were presented. Iodine concentration with skewed distribution are expressed as median and interquartile range (IQR); Mann Whitney and Kruskal-Wallis tests were used for comparing 2 or more groups, respectively. Differences were considered significant when *P* < 0.05. All statistical analyses were carried out using SPSS 13.0 for Windows (SPSS, Inc., Chicago, IL, USA).

## Results

A total of 178 salt samples were collected from different regions of the country, where 92 % (*n* = 164) were packaged salt in which 6 salt samples were from Spanish companies, and 8 % (*n* = 14) were bulk salt. The number of samples collected from urban and rural areas respectively 134 and 44.

All salt samples commercialized by Moroccan companies are labeled "iodized salt" and bearing the logo representative of iodized salt, but 72 % did indicate the adequate level of iodine "30 mg/kg". Two samples from two different companies of 53 identified (3.8 %) mentioned the authorization number of National Food Safety Office (ONSSA: Office National de Sécurité Sanitaire des produits Alimentaires). The date of manufacture/expiry is mentioned in 8 samples out of 164 (4.9 %).

The results of qualitative analysis with the iodine test kit showed that 25 % (45/178) of samples are positive and 75 % (133/178) negative. Besides, 28 % (38/134) of salt commercialized in urban areas is iodized versus 16 % (7/44) in rural areas.

The median iodine concentration of 178 household salt samples was 2.9 mg/kg (IQR: 2.4-3.7). According to Kruskal-Wallis test, there is no significant difference in iodine concentrations between regions (*P =* 0.113) (Table 1).

**Table 1:** Distribution of median and interquartiles ranges of iodine concentration of household salt collected in 12 Moroccan regions

Region	Population households*	Population**	Number of analyzed salt samples	Number of salt samples/100.000 households	Median (IQR) iodine concentration in mg/kg
Region 1: Tanger-Tétouan-Al Hoceima	799 124	3 556 729	16	2	4.7 (3.2–14.5)*
Region 2: L'Oriental	494 530	2 314 346	10	2	2.6 (1.9–3.4)
Region 3: Fès-Meknès	919 497	4 236 892	41	4	2.9 (2.4–3.4)
Region 4: Rabat-Salé-Kénitra	1 015 107	4 580 866	42	4	2.8 (2.1–3.8)
Region 5: Béni Mellal-Khénifra	520 174	2 520 776	4	1	3.6 (2.8–6.5)
Region 6: Grand Casablanca-Settat	1 559 404	6 861 739	18	1	2.5 (2.0–3.3)
Region 7: Marrakech-Safi	928 120	4 520 569	13	1	2.9 (1.8–3.3)
Region 8: Drâa-Tafilalet	277 998	1 635 008	12	4	2.8 (2.4–3.6)
Region 9: Sous-Massa	601 511	2 676 847	11	2	2.4 (2.4–3.2)
Region 10: Guelmim-Oued Noun	90 202	433 757	7	8	2.6 (2.4–3.7)
Region 11: Laâyoune-Sakia El Hamra	78 754	367 758	3	4	4.2 (2.6–4.2)
Region 12: Dakhla-Oued Eddahab	29 385	142 955	1	3	2.9 (2.9–2.9)
Total	7 313 806	33 848 242	178	2	2.9 (2.4–3.7)

*P value = 0.113

**The number of households and population according to the census of the population of Morocco in September 2014. Decree No. 2-15-234 of 19 mars2015 published in the Official Bulletin No. 6358 of May 7, 2015. http://81.192.52.100/BO/fr/2015/BO_6358_Fr.pdf

For samples identified as positive in the qualitative test, the median iodine concentration was 5.8 mg/kg (IQR: 4.2-9.3). For negative samples the median iodine concentration was 2.6 mg/kg (IQR: 2.1-3.4).

For all salt samples, only 4.5 % are considered compliant with legislation (15–40 mg/kg) and the median iodine concentration of these samples was 17.8 mg/kg (IQR: 16.4-21.7) (Table 2).

**Table 2:** Proportion of Households salt samples and their medians and interquartiles ranges of iodine concentration according to iodine concentration intervals mg/kg recommended by the order [13]

	Iodine concentration intervals mg/kg	
	<15 mg/kg	15–40 mg/kg
Number and (%) of Households salt samples	170 (95.5 %)	8 (4.5 %)
Median (IQR) iodine concentration in mg/kg	2.9 (2.4–3.4)	17.8 (16.4–21.7)

Lower median iodine concentration was obtained for salt samples from rural areas compared to that from urban areas, 2.6 mg/kg (IQR: 1.9-3.4) and 2.9 mg/kg (IQR: 2.4-3.7), respectively, but there were no statistically significant difference (*P =* 0.148) (Table 3).

**Table 3:** Sample sizes and medians and interquartiles ranges of iodine concentration in household salt collected from urban and rural areas

Origin of salt sample	Sample size	Median (IQR) iodine concentration, in mg/kg	*P* value
Urban	134 (75 %)	2.9 (2.4–3.7)	0.148
Rural	44 (25 %)	2.6 (1.9–3.4)	
All areas	178 (100 %)	2.9 (2.4–3.7)	

All bulk salts (*n* = 14) are from rural areas, none of was found positive by kit-test. The median iodine concentration in packaged salt is slightly higher than bulk salt, 2.9 mg/kg (IQR: 2.4-3.7) versus 2.5 mg/kg (IQR: 1.5-3.4), but there were no statistically significant difference (*P =* 0.105) (Table 4).

**Table 4:** Sample sizes and medians and interquartiles ranges of iodine concentration in packaged and bulk household salt

Type of salt sample	Sample size	Median (IQR) iodine concentration in mg/kg	*P* value
Packaged salt	164 (92 %)	2.9 (2.4–3.7)	0.105
Bulk salt	14 (8 %)	2.5 (1.5–3.4)	
All salt	178 (100 %)	2.9 (2.4–3.7)	

## Discussion

In the present study, we will evaluate the actual state of salt covertures in Morocco.

Due to constraints of time and resources, we opted for the empirical method of non-probability sampling across the different regions, urban and rural areas.

According to law act n°28–07 of the safety of food products legislation published on 11^th^February 2010, ONSSA became the agency in charge of controlling and monitoring of iodized salt for food. It delivers sanitary authorization for new salt producers, according to a set of specifications before placing to the market. According to our results, only 3.8 % of companies marketing iodized salt have a sanitary authorization. The date of manufacture/expiry is mentioned only in 8 samples out of 164, although it is required by the standards.

None of samples marketed by Spanish companies indicates the level of iodine contained, although according to the regulations, all salts marketed on Moroccan territory must indicate on the packaging "iodized salt" and its concentration. From these results regarding the labeling of salt for food, we note that the producers and the conditions of salt do not meet regulatory requirements. The same thing goes for imported salt or illegally imported salt to Morocco; it does not meet the standards imposed by the law. This represents a real obstacle to the USI.

Analyses of iodine concentration in salt, show that a quarter of households consume iodized salt, which is an improvement compared to a study conducted at Kenitra-Rabat in 1998, where less than of one tenth of salt was iodized [16] and also in relation to the study conducted in the province of Larache in 2003, where less than of one sixth of salt was iodized [18]. Our results confirm the WHO data that rank Morocco among countries where iodized salt consumption is less than 35 % [22]. However, less than one twentieth was properly iodized (15–40 mg/kg).

The proportion of iodized salt is higher in urban areas than in rural areas. This difference is partially explained by the use of bulk salt in rural areas. A non-negligible proportion of the samples collected come from bulk salt purchased at retail. This bulk salt which in addition is non-iodized was used by almost a third of rural households.

According to tests used in data analysis, there are no significant differences in iodine concentrations between regions, between urban and rural areas and between packaged and bulk salt.

This shows that Morocco is still far from reaching the goal of broad coverage of iodized salt. This should draw the attention of public health authorities about the progress of the USI strategy implementation, especially when this is about to celebrate its 20th anniversary on 2015.

One of the lessons learned from countries that have managed to eliminate or substantially reduce IDD is the awareness of the key role that continuous monitoring of the quality of salt and monitoring population iodine status in the effectiveness of iodization programs. Proper monitoring would all for better adjustment of intervention and assessment of impact. As they allow you to adjust the intervention and assess its impact.

However, few are the countries that have a reliable surveillance system [4].

The National Iodine Deficiency Disorders Control Program can achieve its objectives if an internal monitoring and external quality control program is put into place as well as planning additional operational strategies like. Governmental and salt producers must work in partnership and help small producers to gather in cooperatives. Moreover, ensuring the availability of potassium iodate and test-kits and putting at the disposal of salt producers. The control and monitoring of dietary salt should be strengthened to prohibit the sale of bulk salt in traditional markets and rural areas. Health authorities need to adopt a different strategy for people who have no access to iodized salt such as iodized oil, especially in the most affected areas where the prevalence and severity of IDD is highly amounted and to regularly monitor iodine intake in the population, using an assay of urinary iodine.

## Conclusion

Since 1995 Morocco has adopted a National Iodine Deficiency Disorders Control Program to fight against IDD by introducing iodized salt and enacting legislation and standards to ensure the sustainability of the program. 20 years after implementation of the USI program, our study shows that the goal of a widespread use of iodized salt is still far from being achieved. In 2015, 25 % of Moroccan households use iodized salt, but the proportion of iodized salt conforming to the regulation standards is only 4.5 %; with a median iodine concentration of 17.8 mg/kg (IQR: 16.4-21.7). The salt commercialized by foreign companies does not meet the Moroccan standards, which represents a risk to the health of the population. On the other hand, the use of bulk salt by 8 % of households is a major obstacle to the USI. Effective surveillance systems should include monitoring of iodine content of salt at industrial, retail and household levels to ensure that iodization program is safe and effective.

### Open access

This article is distributed under the terms of the Creative Commons Attribution 4.0 International License (http://creativecommons.org/licenses/by/4.0/), which permits unrestricted use, distribution, and reproduction in any medium, provided you give appropriate credit to the original author(s) and the source, provide a link to the Creative Commons license, and indicate if changes were made. The Creative Commons Public Domain Dedication waiver (http://creativecommons.org/publicdomain/zero/1.0/) applies to the data made available in this article, unless otherwise stated.

## Abbreviations

IDD: Iodine Deficiency Disorders
IQR: interquartile range
ONSSA: Office National de Sécurité Sanitaire des produits Alimentaires (National Food Safety Office)
ppm: Parts-per-million
USI: Universal Salt Iodization
WHO: World health organization
